# Predictors of post neonatal mortality in Western Kenya: a cohort study

**DOI:** 10.11604/pamj.2018.31.114.16725

**Published:** 2018-10-15

**Authors:** Grace Kaguthi, Videlis Nduba, Martien Wilhelm Borgdorff, Suzanne Verver

**Affiliations:** 1 Kenya Medical Research Institute, Centre for Respiratory Diseases Research, Nairobi, Kenya; 2 Academic Medical Centre, University of Amsterdam, the Netherlands; 3 Department of Public Health, Erasmus Medical Centre, Rotterdam, the Netherlands; 4 Dutch Tuberculosis Foundation, the Hague, the Netherlands

**Keywords:** Mortality, post-neonatal, tuberculosis, infant, vaccine

## Abstract

**Introduction:**

to determine the predictors of mortality in infants in Siaya, western Kenya, ahead of novel tuberculosis (TB) vaccine trials in the same population.

**Methods:**

in a study to determine tuberculosis incidence, 2900 infants aged 0-45 days, weighing ≥ 1700g were enrolled. Four monthly follow up visits were conducted for at least 12 months. HIV testing was done at six weeks of age. Free ancillary care was provided. Deaths were reported by parents, study staff and community workers. Cox proportional Hazard analysis was used to identify risk factors. The period of analysis commenced at six weeks old and was censored at 12 months of age.

**Results:**

included in the analysis were 2528 infants with 2020 person years of follow up (pyo). There were 117 deaths (4.6 %). The post-neonatal mortality rate was 58 (95% CI: 48, 69) per 1000 pyo. In multivariate analysis, health facility births were protective against mortality (Hazard Ratio (HR) 0.54; 95% CI: 0.34, 0.84) and infant HIV infection at baseline was associated with increased mortality (HR 10.3; 95% CI: 6.40, 16.7). HIV uninfected infants born to HIV infected mothers had increased hazards of mortality (HR 1.73; 95% CI: 1.03, 2.90). Gender, weight at six weeks, maternal education and occupation were not significant predictors of mortality.

**Conclusion:**

infant mortality was high and was associated with being born outside a health facility, maternal HIV infection and HIV infection of the infant. Measures to decrease mother to child transmission and other HIV control measures need to be strengthened further to see incremental reductions in infant mortality.

## Introduction

Strategies to reduce child deaths have been extensively studied [1-3]. Over the last two decades considerable progress has been made in averting thousands of child deaths [4]. This is attributable to improvements in socio-economic conditions and scale up of disease specific interventions such as use of insecticide- treated bed nets, oral rehydration therapy for diarrheal disease targeting the main causes of childhood deaths. In fact, Kenya has experienced reduction of infant mortality from 77/1000 in 2003 [5] to 52/1000 live births in 2008 [6] and 39/1000 live births in 2014 [7]. Despite this considerable decline from high levels of mortality, it is a forty-fold higher probability of death compared to a child born in developed countries where the chances are less than one death in every one thousand live births [8]. Several studies have revealed various determinants of child mortality. Child spacing, birth order, maternal illiteracy, income disparities, rural versus urban residence, relative distance from health facilities have all been known to account for high child mortality [9-11]. These have been identified from cross-sectional studies such as national health and demographic surveys [1, 2]. Data from cohort studies have been less frequently employed although they might provide a better assessment of relationships between possible causal factors and mortality [12]. Further, due to close follow up in cohort studies, more information on proximal health variables may be available. We conducted a cohort study between June 2009 and September 2011 to determine what factors predicted infant mortality in Western Kenya. This was a preparatory study to obtain tuberculosis (TB) incidence estimates amongst infants in preparation for trialing novel TB vaccine candidates in the same population, with infant mortality as a secondary objective. Deaths that occur in the conduct of a clinical trial may be misattributed to the investigational agent if background mortality rates and causes are not clear. Excess mortality also implies missed end-points when they occur prematurely.

## Methods

### Setting

The study area was predominantly rural in Karemo, Gem and Boro Divisions of Siaya County, Western Kenya. The infant mortality rate at the time in this area was 110/1000 live births [13], more than double the national average. The area has a high prevalence of the HIV and TB syndemic, with perennial intense malarial transmission [13, 14]. About 80% of women deliver at home without the help of a skilled attendant [15, 16]. Subsistence farming is the main economic activity. Educational attainment of female population is low: 9.3% of women have some secondary education while 49.3% have some primary education. Siaya County is served by 31 dispensaries and health facilities, manned by nurses who provide vaccinations, antenatal care, contraception, and empiric treatment of basic ailments. Several of these facilities provide Anti-Retroviral Therapy at Patient Support Centers (PSCs). Hospitalizations mainly occur at the Siaya County Hospital. The proportion of low birth weight among neonates weighed at birth (53%) is 6% in the study area [17].

### Study population

The target was to enroll a total of 2900 infants 0-45 days of age, between June 2009 and June 2010 who were at least 1700g at enrolment. Follow up visits were carried out every four months for at least one year, maximum of two years depending on the time of enrolment. Participants enrolled earlier had a longer duration of follow up. Visits were conducted at a health facility of the parent's choice or at home. To enroll infants, we received birth notifications from community health workers who frequently doubled as Traditional Birth Attendants (TBAs). Study nurses on motor bikes visited homes of interested potential participants. They obtained informed consent, took anthropometric measurements and administered BCG vaccine to those unvaccinated. Infants below 1700 g at time of enrolment were excluded. At age six weeks, Deoxy-ribo-Nucleic Acid (DNA) Polymerase Chain Reaction (PCR) HIV testing (COBAS^®^ HIV-1 Amplicor by ROCHE) was offered to all participants. Additional HIV testing was done as part of TB investigations for those with presumptive TB (at time of study were named TB suspects), during follow up and at study exit. Parents and participants who tested HIV positive, were referred to the Patient Support Centre for Anti-Retroviral Therapy (ART) initiation and care. A dedicated study nurse visited these participants to counsel on feeding options and encourage ART initiation. Free ancillary care was provided at the study clinic. The study covered the cost of transport up to 5 USD and hospitalizations at the Siaya County Hospital. Supplemental feeds provided by UNICEF to the hospital were also administered by the study nutritionist for participants who met the criteria, according to the WHO guidelines for management of malnutrition. Deaths were notified by parents, study staff and community health workers. The families of participants who died at home or at facilities outside the County Hospital were visited by a study nurse to obtain ante-mortem history from the primary caregiver using a semi-structured questionnaire. At each visit, data was entered electronically into a customized Microsoft 2009 SQL^®^ database with edit checks and validations. The predetermined sample size was powered to identify incident tuberculosis cases, with mortality incidence as a secondary objective.

### Ethical considerations

The study was approved by the Kenya Medical Research Institute Ethics Committee (SSC Number 1465), and conducted according to International Committee for Harmonization of Clinical Trials-Good Clinical Practice (ICH-GCP). Written Informed consent was obtained from mothers prior to initiating study procedures. Study Numbers were assigned to maintain privacy and confidentiality. Access to study records was limited to study staff and monitors.

### Cause of death ascertainment

To obtain the causes of death for post-neonatal mortalities, two medical officers reviewed available morbidity data from the study clinic, admission notes and investigations as well as the ante-mortem history for deaths that occurred at home. Immediate and underlying causes of deaths were independently assigned by each medical officer using the International Classification of Diseases-10 (ICD-10). Disagreements were resolved by consensus. Anthropometric data on weight for age was available from follow up visits or unscheduled visits. Weight for age Z-Scores from the World Health Organisation Charts were used to define presence of under-nutrition [18]. Weight of two or more standard deviations from the median for age, (≥-2SD) was classified as moderate to severe malnutrition. Weight for age Z-scores of greater than one, up to three months before death, were analysed as potential underlying risk factor of death.

### Statistical analysis

To minimise survival bias introduced by variable study entry which could have been related to neonatal deaths (left truncation), the analysis time began when infants were six weeks of age and above. Follow-up time was censored at 12 months of age. Person-time was calculated from 6 weeks of age till 12 months of age, death, or loss to follow-up, whichever occurred first. Descriptive statistics were used to describe demographic and clinical characteristics, causes of death and infant mortality rate. Unadjusted and adjusted hazard ratios for mortality were computed using Cox proportional hazards analysis. Logistic regression was used in bivariate analysis to evaluate the relationship between selected predictors. Missing data was excluded.

## Results

**Figure 1:**
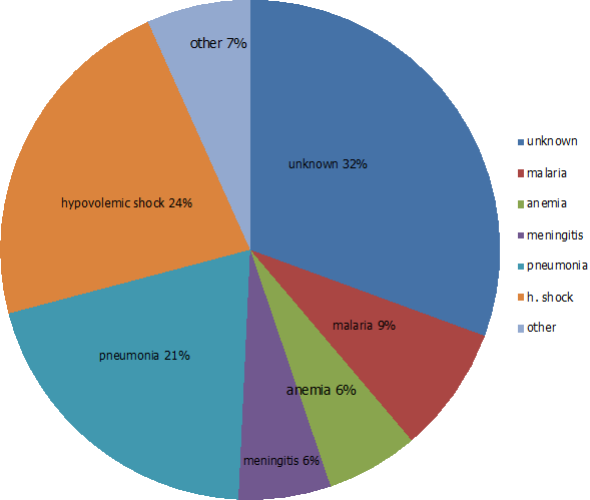
immediate causes of death (n = 88/117)

**Figure 2:**
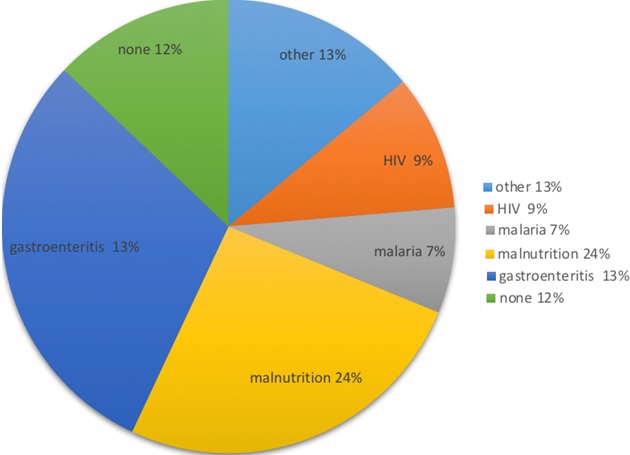
underlying causes of death (n = 110/117)

In 2010, there were 3534 registered births in the study area [19]. The study received 3538 birth notifications between June 2009 and June 2010. Of these, 2900 (82.1%) were enrolled. There were 46/2900 (1.6%) deaths between birth and six weeks and 326/2900 (11%) were lost to follow up at six weeks. Therefore, 2528 infants, six weeks old and above were included in this analysis (Table 1). Those included had together 2020 total person years of follow up. Average person-time was 0.8 person-years. At twelve months, cumulative Loss to Follow Up (LTFU) was 20% (Table 1). There were 117 (4.6%) deaths, between six weeks and twelve months of age. The post- neonatal mortality rate was 58 per 1000 person years of follow up (95% C.I 48, 69). The post neonatal mortality ratio was 46 per 1000 live births. Four in ten deaths occurred at health facilities. Patients with sick visits at the study clinic were more likely to be hospitalized (HR 2.55; 95% CI: 1.43, 4.57). Immediate causes of death were identifiable in 88/117 (75%) cases. Of these, the most frequently identified causes of death were: hypovolemic shock/dehydration (30%), pneumonia (26%), malaria (10%) and anemia (9%). Together these accounted for 66/88 (66%) of identifiable deaths (Figure 1). Of the 36/117 (31%) cases for which an immediate cause of death could not be assigned, 29 cases had inconclusive information, and seven had no information at all regarding the ante-mortem circumstances. Regarding the underlying causes of death, 110/117 (94%) had information derived from clinical notes on co-morbidities which were underlying causes of death. About half of the underlying causes of death were attributed to undernutrition 24/110 (22%), diarrheal disease 17/110 (16%) and anemia 13/110 (12%). Other frequent underlying causes were infant HIV infection and malaria (Figure 2).

**Table 1:** enrolment and follow up

Number of participants (n)	Age	Deaths (n)	Loss to Follow Up (LTFU)
enrolled 2900	0 to 6 weeks	47	LTFU* before 6 weeks : 325/2900(11%)
2528 survivors (included in this analysis)	>6 weeks to ≤ 4 months	46	Total LTFU: 626/2900 (21%)
	>4 months to ≤ 8 months	47	
	8 months ≤ 12 months	24	

In the univariate analysis of predictors of mortality, infants born in health facilities had lower hazards of mortality, compared to home deliveries (Hazard Ratio (HR) 0.53; 95% CI: 0.35, 0.82). This was also observed in the multivariate analysis (adjusted HR 0.54; 95%CI: 0.34, 0.84). Mothers who had secondary education and above were three times more likely to deliver in a health facility. OR 3.02 (95% CI: 2.38, 3.84). A high proportion of HIV positive mothers (16%) transmitted the virus to their infants and tens of these infants 26/71 (36%) of the infants tested at six weeks, died before HIV results could be disclosed. Infants who were HIV infected were ten times more likely to die than uninfected infants (adjusted HR 10.3; 95% CI: 6.40, 16.7). HIV uninfected infants born to HIV infected mothers were twice as likely to die compared with their counterparts born to uninfected mothers (adjusted HR 1.72; HR 95% CI: 1.03, 2.90). All these effects were consistently observed in the adjusted and unadjusted models (Table 2). Infants whose weight for age category was one standard deviation or more from median (WHO growth charts 2009), had four times the rates of mortality as peers who had normal weight for age Z score. (HR 4.02; 95% CI (1.28, 12.6). However, weight for age Z score was not a statistically significant predictor in the adjusted model aHR (2.81; 95% CI: 0.88, 8.96) Maternal occupation and education and infant gender were not shown to be associated with infant survival. However, mothers with secondary education and above were 32% less likely to attend sick visits at the clinic OR (0.68 95%CI: 0.50, 0.94), and their infants were 30% less likely to be hospitalized OR (0.70 95% CI: 0.55, 0.90). Maternal occupation did not significantly impact the choice of home or health facility birth, sick visits nor hospitalisations.

**Table 2:** predictors of one year post-neonatal mortality

Variable	Total number of infants	Number of deaths	person years of follow up	mortality rate/1000 pyrs	Unadjusted Hazard Ratio (95% CI)	Adjusted Hazard Ratio (95% CI)
Total	2528	117	2020	58		N=2440
**Gender**						
Male	1320	66	1037	64	1 (ref)	1(ref)
Female	1224	51	983	52	0.82 (0.57-1.18)	0.87 (0.60-1.27)
**Place of Birth**						
Home	1636	91	1308	70	1 (ref)	1(ref)
Health Facility	876	26	699	37	0.53 (0.35-0.82)	0.54 (0.34-0.84)
Missing	16	0	-	-	-	-
**Mother’s occupation**						
Not working	1408	67	1110	60	1 (ref)	1(ref)
Working	1079	48	878	55	0.91 (0.63-1.32)	0.99 (0.68-1.44)
Unknown	41	2	-		-	-
**HIV status**						
Mother HIV negative/Infant HIV negative	2101	75	1681	43	1 (ref)	1(ref)
Mother HIV positive/infant HIV negative	316	19	260	73	1.70 (1.02-2.81)	1.72 (1.03- 2.90)
Infant HIV positive	71	23	46	506	11.1 (6.93-17.7)	10.3 (6.40-16.7)
Unknown	40	0	-		-	-
**Mother’s education**						
None/Primary	2174	105	1748	60	1(ref)	1(ref)
≥Secondary	326	10	251	40	0.66 (0.34-1.25)	0.88 (0.45-1.69)
Missing	28	2			-	-
**Weight for age at 6 week**						
None to -1SD	2509	114	2007	57	1(ref)	1(ref)
Moderate-Severe (≥-2SD)	19	3	13	230	4.02 (1.28-12.64)	2.81 (0.88- 8.96)

## Discussion

The post-neonatal mortality ratio in the study area was 46 (95% CI: 39, 56) per 1000 live births and is comparable to estimates by the Health and Demographic Surveillance System in the general study area at the time of the study (52/1000 live births) [20]. Our analysis included infants who survived to six weeks of age, rather than to four weeks, hence the lower mortality ratio may be an underestimate. The post-neonatal mortality rate (58 (95%CI: 48, 69) per 1000 pyo is higher than a similar study in Eastern Uganda, which found 40 per 1000 pyo [21], Eastern Uganda has a less intense malarial transmission [19-22] and antenatal HIV prevalence rates are less than half of those in Western Kenya [23]. After controlling for gender, maternal education and occupation, health facility births were significantly protective against mortality and infant and maternal HIV infection was associated with increased hazards of mortality. Unlike previous surveys, we did not find maternal occupation or education to significantly predict mortality. Infants of mothers with secondary education attended fewer sick visits and had less frequent hospitalisations, therefore, the inability to demonstrate significant mortality benefit could be due to the small numbers of these women in the study. We found factors of morbidity such as HIV status, health access and utilization (health facility births) to predict mortality at one year of age. This could be due to the fact that the study population was relatively homogenous with respect to these socio-demographic indices. Alternatively, maternal education and occupation might be intermediate factors of mortality that drive health facility use and are less significant in the presence of more direct measures.

### HIV infection

Infant HIV infection was negatively associated with survival. A high proportion of HIV positive mothers, (16%), in line with a contemporaneous national survey [24] transmitted the virus to their infants and tens of these infants, and (36%) of the 71 HIV infected babies, died before HIV results could be disclosed, implying that interventions after transmission has occurred appear to have limited influence on mortality. Fortunately, it appears several interventions to reduce Mother To Child Transmission (MTCT) are reversing the devastating trend of transmission and HIV mortality [25, 26].

### Maternal HIV infection

More recently, these declines in MTCT have turned the focus to HIV exposed uninfected (HEU) infants who tested HIV negative but were born to HIV infected mothers who have been shown to have higher respiratory and diarrheal disease morbidity, more frequent hospitalisations, impaired immunity and growth [27, 28]. Our study tested infants at six weeks, upon suspicion of tuberculosis, and at study close out. Mothers were encouraged and supported to initiate ART, and safe feeding practices. We were able to show HEUs had higher hazards of mortality at one year, compared to HIV uninfected peers born to HIV negative mothers despite these measures. Similar outcomes have been observed in smaller studies in Malawi, Uganda [29-31]. Additional effects may be mediated by catastrophic health costs incurred in treatment of opportunistic infections which interferes with food security and health care access [32]. A sick mother is also unlikely to optimally care for her baby. Having shown these infants to have increased hazards of mortality, assuring maternal health by motivating early initiation into ART and continued compliance could provide the most unified approach in reducing their vulnerability.

### Health facility utilization

Health facility deliveries have been known to be protective against perinatal mortality [33, 34]. We could not find studies showing medium to long term effects of health facility deliveries. Our study showed that one year mortality was significantly lower for infants born in health facilities compared to those born at home. Mothers who gave birth in health facilities may have been more likely to use facilities in the future for immunization or seeking care for illnesses, leading to a longer term mortality benefit.

### Causes of death

Similar to verbal autopsy reviews of under-five mortality during a similar time period in the same study area [35], the majority (60%) of identifiable causes of death were due to severe dehydration following gastro-enteritis, pneumonia, malaria and anemia. There were slight methodological differences between that study and ours due to availability of more detailed diagnostic and clinical information to our study team. Under-nutrition was identified as underlying cause of death in a third of mortalities. Any degree of under-nutrition is known to increase mortality risk [3]. It is frequently underreported as a cause of death, and therefore this proportion may even be higher. Under-nutrition is known to be a potentiating cause of death in up to 53% of deaths in countries in the developing world [36]. In addition to contributing to excess morbidity, hospitalization and mortality, under-nutrition has irreversible long term effects on cognitive development, individual earnings and economic growth [37].

### Strengths and Limitations

Correlates of infant mortality are frequently predicted from surveys and ecological studies [38, 39]. Cohort studies are infrequently done. We accessed and followed up a large cohort of infants. Another strength is that the study recruited the large majority (82%) of the neonates born at that time in the study area. Therefore, we consider the findings largely generalizable to similar settings in Sub-Saharan Africa. The study has several limitations. By design, very low and extremely low birth weight infants were excluded, possibly under-estimating mortality. We experienced loss to follow up (LTFU) of 21%). More than half of this occurred at or before six weeks. This is cultural, closer to delivery pregnant women in the study area migrate to be closer to female relatives in proximity and return to their homes thereafter. LTFU thereafter was 10%, we therefore consider that this did not significantly impact our estimates, as our post-neonatal mortality rate is in line with separate surveys of the study area at the same time. Nevertheless it is plausible that exclusion of the early infancy period, we inadvertently selected for those with lower risk of mortality. Further, those who were lost to follow up could have different health seeking behavior and therefore higher mortality risk. This is unlikely to have shifted the results as it was less than 10% for the period under consideration.

## Conclusion

We have shown that infant HIV infection and exposure significantly increased mortality among infants and that factors of health utilization such as health facility births reduced the hazards of mortality. Since then the infant mortality rate in the study area decreased from 52 to 35 deaths per 1000 [40]. There is a decline in most of the childhood death indicators in Kenya and the East African Region, possibly due to introduction of pneumococcal vaccination in 2011, a leading cause of morbidity and mortality in low income countries with high infant mortality [1, 41, 42], as well as scale up of ART for infected persons. Our study shows other areas where gains in survival can be improved, such as facilitating health facility use and access and scaling up ART among persons living with HIV. Ultimately, sustaining and improving on gains in child survival in developing countries will call for societal change [43] to address global and national economic inequities.

### What is known about this topic

- Regional infant mortality is high and is likely to confound novel vaccine trials of malaria and tuberculosis in the study area;
- A majority of mothers (80%), deliver outside of a health facility, yet health facility births are protective against neonatal mortality;
- HIV infected infants have high mortality compared to HIV uninfected peers.

### What this study adds

- HIV uninfected infants born to HIV infected mothers have two times more likely to die in the first year of birth compared to peers born to HIV negative mothers;
- Health facility access and utilization at birth seems increases survival to one year;
- Detailed anthropometric data gathered over time show under-nutrition is a significant potentiating cause of death, despite provision of supplemental feeds and close monitoring.

## Competing interests

The authors declare no competing interests.
